# Oxytocin receptor binding sites in the periphery of the neonatal mouse

**DOI:** 10.1371/journal.pone.0172904

**Published:** 2017-02-24

**Authors:** Maria A. Greenwood, Elizabeth A. D. Hammock

**Affiliations:** 1 Program in Neuroscience, The Florida State University, Tallahassee, FL, United States of America; 2 Department of Psychology, The Florida State University, Tallahassee, FL, United States of America; University of Texas at El Paso, UNITED STATES

## Abstract

Oxytocin (OXT) is a pleiotropic regulator of physiology and behavior. An emerging body of evidence demonstrates a role for OXT in the transition to postnatal life of the infant. To identify potential sites of OXT action via the OXT receptor (OXTR) in the newborn mouse, we performed receptor autoradiography on 20 μm sagittal sections of whole postnatal day 0 male and female mice on a C57BL/6J background using the ^125^iodinated ornithine vasotocin analog ([^125^I]-OVTA) radioligand. A competitive binding assay on both wild-type (WT) and OXTR knockout (OXTR KO) tissue was used to assess the selectivity of [^125^I]-OVTA for neonatal OXTR. Radioactive ligand (0.05 nM [^125^I]-OVTA) was competed against concentrations of 0 nM, 10 nM, and 1000 nM excess unlabeled OXT. Autoradiographs demonstrated the high selectivity of the radioligand for infant peripheral OXTR. Specific ligand binding activity for OXTR was observed in the oronasal cavity, the eye, whisker pads, adrenal gland, and anogenital region in the neonatal OXTR WT mouse, but was absent in neonatal OXTR KO. Nonspecific binding was observed in areas with a high lipid content such as the scapular brown adipose tissue and the liver: in these regions, binding was present in both OXTR WT and KO mice, and could not be competed away with OXT in either WT or KO mice. Collectively, these data confirm novel OXT targets in the periphery of the neonate. These peripheral OXTR sites, coupled with the immaturity of the neonate’s own OXT system, suggest a role for exogenous OXT in modulating peripheral physiology and development.

## Introduction

Oxytocin (OXT) plays an influential role in mammalian adult behavior and physiology, including but not limited to maternal experiences such as parturition, milk let-down, and mother-infant bonding [1, 2]; social recognition and communication [3, 4]; and social bonding [5, 6]. Genetic association studies in humans [7] as well as experimental treatments with intranasal OXT [8] both provide evidence for a role for OXT in human social behavior. However, rodents have long served as excellent model systems of the neurobiology of social behavior, especially in regard to the role of OXT in maternal behaviors [9]. While much is known about the function of OXT within the adult brain, less is known about the mechanistic role of OXT in infant development.

Perinatal exposure to single doses of OXT has both acute and long-term effects on physiology and behavior [10–12]. For example, OXT regulates GABA signaling in the neonate to dampen neuronal excitability during delivery and offer protection from potential hypoxia [13–16]. Additionally, single subcutaneous injections of OXT rescue a lethal feeding defect and adult behavioral abnormalities in *Magel2* deficient mice [17, 18]. Despite the evidence of a role for OXT in the newborn mouse, there are not enough data to ascertain where endogenous or exogenous OXT may act throughout the neonate. We previously reported preliminary evidence of OXT receptor (OXTR) expression in several peripheral tissues of the mouse embryo [19]. In the current study, we determined the specificity of OXTR ligand binding in several selected regions throughout the periphery of the neonate on postnatal day 0 (P0). In newborn male and female mice, we determined the specific ligand binding pattern of OXTR using receptor autoradiography, by comparing ligand binding data in wild-type and OXTR knockout mice with and without competitive binding for OXTR. Understanding the specific expression pattern of OXTR in the newborn mouse could aid the identification of novel regions of interest for the effects of either endogenous OXT or socially-acquired exogenous OXT on neonatal development.

## Materials and methods

### Mice

*Oxtr* mice (*Oxtr*^*tm*1.1*Knis*^) [20] were bred within our laboratory after genetically-confirmed backcrossing to C57BL/6J (Speed Congenic Service, Jackson Laboratories) [19]. All procedures were performed after approval by the Institutional Animal Care and Use Committee of Florida State University (protocols 1425 and 1446) in accordance with state and federal guidelines (Guide for the Care and Use of Laboratory Animals of the National Institutes of Health). Pregnancies were not timed; heterozygous breeding pairs were checked daily, with the first appearance of a litter established as postnatal day 0 (P0). On P0, parents were removed from the homecage, litters were euthanized under prolonged CO_2_ exposure, tail samples were collected, and whole body tissue was frozen in liquid nitrogen. Specimens were stored at -80°C until cryosectioning. All offspring were born of heterozygous breeder pairs, collected from multiple litters.

### Genotype and sex determination

Tail samples were used to determine sex and *Oxtr* genotype (wild-type or knockout) of the neonates, via PCR using established methods [19, 21]. To determine genetic sex, the forward primer (5′-ccgctgccaaattctttgg-3′) and the reverse primer (5′-tgaagcttttggctttgag-3′) generated a 290 bp product from the *Smcy* gene on the Y chromosome, and a 330 bp product from the *Smcx* homolog on the X chromosome under the following thermal cycling conditions: 95°C for 7 min; 35 cycles of 93°C for 30 s, 58°C for 30 s, 72°C for 30 s; 72°C for 10 min. *Oxtr* genotypes were determined by PCR with the forward primer (5’-ctggggctgagtcttggaag-3’) and the reverse primers for wild-type (5’-ctcgatactccagttggctgc-3’) or knockout (5’-gttgggaacagcggtgatta-3’). These primers generated a 665 bp product for the wild-type allele, a 450 bp product for knockout allele under the following thermal cycling conditions: 94°C for 5 min; 37 cycles of 94°C for 30 s, 57°C for 45 s, 72°C for 90 s; 72°C for 7 min.

### Receptor autoradiography

Tissue was cryosectioned in 8 series at 20 μm in the sagittal plane and mounted on SuperFrost Plus slides. Sections were stored at −80°C until used in the receptor autoradiography protocol. Receptor autoradiography was performed by standard methods [19] using 0.05 nM ^125^I labeled OXT receptor ligand (iodinated-ornithine vasotocin analog NEX254 [OVTA]; [22, 23]; [^125^I]-OVTA, Perkin-Elmer, Waltham, MA). To assess nonspecific tissue binding in the periphery of the neonatal mouse, unlabeled OXT peptide (oxytocin acetate salt hydrate, cat# O6379, Sigma-Aldrich, St. Louis, MO) was added to the radioactive tracer in concentrations of 0 nM, 10 nM, and 1000 nM separately on sets of adjacent slides. Increasing concentrations of unlabeled OXT peptide were competed with ^125^I-OVTA for receptors, to decrease the visible signal in regions containing OXTR. This competition was performed in both OXTR wild-type and knockout mice. Autoradiographic films (Kodak Biomax MR film, Carestream Health, Inc., Rochester, NY, USA) were exposed to slides and ^14^C autoradiographic standards (ARC-0146; American Radiolabeled Chemicals, St. Louis, MO, USA) for approximately 72 hours before developing (Mini-Medical/90 X-ray film processor, AFP Imaging, New York).

### Image analysis

After autoradiography, all slides were post-processed with cresyl violet stain for unbiased region of interest measurements. Briefly, slides were incubated in 0.5% cresyl violet solution at 37°C for 5 minutes, rinsed in distilled water, differentiated in 95% ethanol alcohol in two five-minute washes, and dehydrated in 100% ethanol alcohol in two five-minute washes. Finally, slides were cleared in CitriSolv (Decon Labs Inc., King of Prussia, PA) in two five-minute washes and cover-slipped. Films and stained slides were scanned on a flatbed scanner at 1200 dpi (EPSON, Epson Perfection V600 Photo). Regions of interest were identified on post-processed slides and then measurements were collected from corresponding film images. Quantifications were recorded in ImageJ (NIH, Bethesda, MD) using the brush-selection tool, from three consecutive sections within each animal. Local background values were obtained from non-tissue background of the slide adjacent to the region of interest and measured in a 20x20 pixel region, then subtracted from the region of interest values to generate local densitometry values. Ligand binding values in μCi/gram were calculated by interpolation “interp1,” MatLab 8.1.0 (TheMathworks, Natick, MA, USA) to the linear range of the ^14^C autoradiographic standard on the same film [24]. For quantification, no image adjustments were made with the exception of image inversion so that higher numbers represented more dense binding. Composite images were created using the TurboReg [25] plugin for ImageJ using the rigid-body alignment algorithm. For pseudocolor composites, the autoradiography images were adjusted for brightness to minimize the appearance of the film background.

### Statistical analysis

All quantified regions were analyzed by Multivariate Analysis of Variance (MANOVA) for main effects of genotype, competition dose, and genotype x dose interactions using IBM SPSS Statistics 22. Individual regions of interest were analyzed to determine if they had specific OXTR binding by ANOVA with Bonferroni correction for multiple comparisons. Our sample sizes included Male wild-type (WT) (n = 3), Male knockout (KO) (n = 3), Female WT (n = 3), Female KO (n = 1). This study is underpowered to detect modest sex differences and therefore, sex was not included as an independent variable in our statistical analysis. Absence of specific signal in the OXTR KO as well as dose-dependent displacement with the unlabeled competitive ligand only in OXTR WT neonates was our a priori threshold for determining specific OXTR ligand binding. This would be reflected in a significant statistical interaction between genotype and competition dose.

## Results

**Fig 1:**
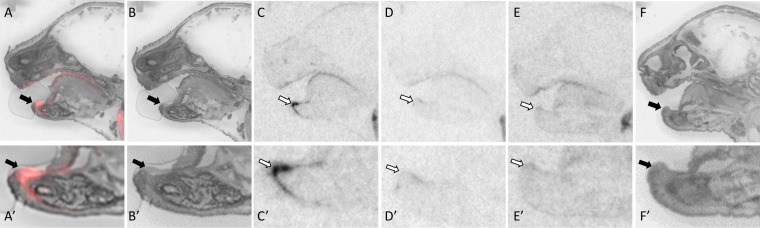
OXTR ligand binding in the primordial tooth of a P0 male mouse. Pseudo-color composite (OXTR in red, cresyl violet counterstain in gray) (A), followed by cresyl violet counterstain (B), with black arrows indicating region of interest. OXTR ligand binding is indicated by white arrows in the 0 nM OXT competition (C), followed by 1000 nM OXT competing with OVTA radioligand to reduce the signal (D). OXTR ligand binding was not present in OXTR KO mice (E), followed by the post-processed cresyl violet tissue of the OXTR KO (F).

**Fig 2:**
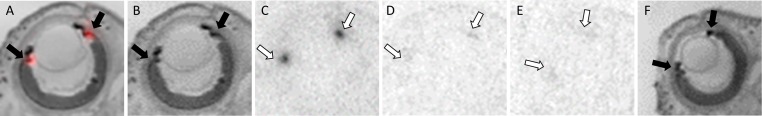
OXTR ligand binding in the eye of a P0 male mouse. Pseudo-color composite (OXTR in red, cresyl violet counterstain in gray) (A), followed by cresyl violet counterstain (B), with black arrows indicating region of interest. OXTR ligand binding is indicated by white arrows in the 0 nM OXT competition (C), followed by 1000 nM OXT competing with OVTA radioligand to reduce the signal (D). OXTR ligand binding was not present in OXTR KO mice (E), followed by the post-processed cresyl violet tissue of the OXTR KO (F).

**Fig 3:**
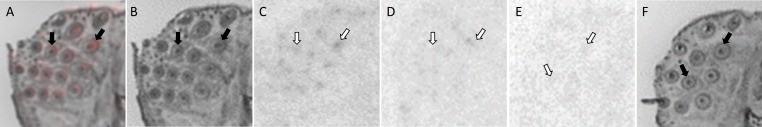
OXTR ligand binding in the whisker pads of a P0 male mouse. Pseudo-color composite (OXTR in red, cresyl violet counterstain in gray) (A), followed by cresyl violet counterstain (B), with black arrows indicating region of interest. Ligand binding specificity was restricted to follicles. OXTR ligand binding is indicated by white arrows in the 0 nM OXT competition (C), followed by 1000 nM OXT competing with OVTA radioligand to reduce the signal (D). OXTR ligand binding was not present in OXTR KO mice (E), followed by the post-processed cresyl violet tissue of the OXTR KO (F).

**Fig 4:**
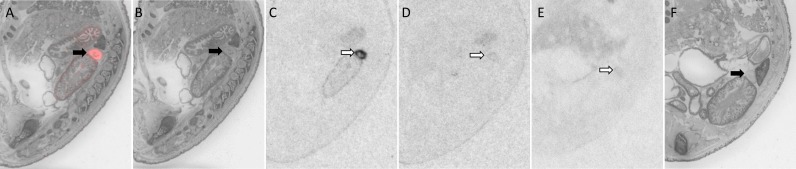
OXTR ligand binding in the adrenal gland of a P0 male mouse. Pseudo-color composite (OXTR in red, cresyl violet counterstain in gray) (A), followed by cresyl violet counterstain (B), with black arrows indicating region of interest. OXTR ligand binding is indicated by white arrows in the 0 nM OXT competition (C), followed by 1000 nM OXT competing with OVTA radioligand to reduce the signal (D). Specific binding can be observed on the kidney (C) just below the adrenal gland, and this is also competed away by 1000 nM OXT (D). Nonspecific binding to lipids in the duodenum can be seen above the adrenal gland; this faint signal does not change across competition conditions. OXTR ligand binding in the adrenal gland (or kidney) was not present in OXTR KO mice (E), followed by the post-processed cresyl violet tissue of the OXTR KO (F).

**Fig 5:**
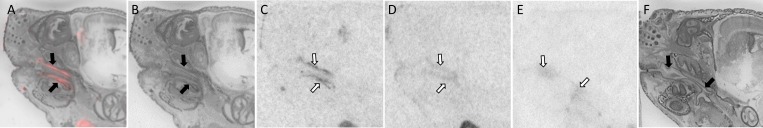
OXTR ligand binding in the periodontium of a P0 male mouse. Pseudo-color composite (OXTR in red, cresyl violet counterstain in gray) (A), followed by cresyl violet counterstain (B), with black arrows indicating region of interest. OXTR ligand binding is indicated by white arrows in the 0 nM OXT competition (C), followed by 1000 nM OXT competing with OVTA radioligand to reduce the signal (D). OXTR ligand binding was not present in OXTR KO mice (E), followed by the post-processed cresyl violet tissue of the OXTR KO (F).

**Fig 6:**
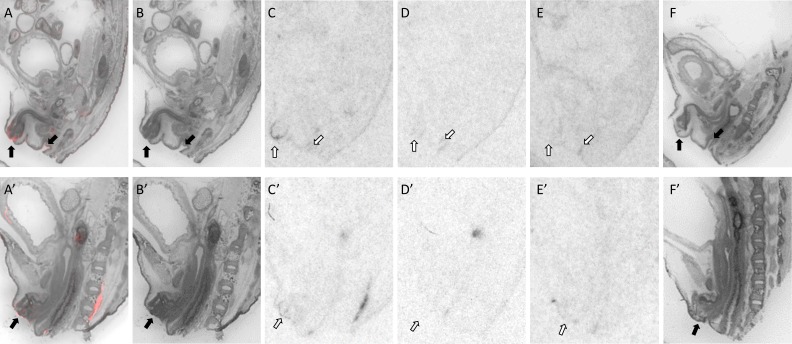
OXTR ligand binding in the anogenital region of P0 mice. A male is depicted in A-F, a female in A’-F’. Pseudo-color composite (OXTR in red, cresyl violet counterstain in gray) (A, A’), followed by cresyl violet counterstain (B, B’), with black arrows indicating region of interest. OXTR ligand binding is indicated by white arrows in the 0 nM OXT competition (C, C’), followed by 1000 nM OXT competing with OVTA radioligand to reduce the signal (D, D’). Specific binding can be observed in the female mouse (C’), with part of the spinal cord (cauda equina) displaying robust signal (not quantified) that diminishes with increasing OXT competition (D’). Nonspecific binding can be seen more central to the image in female mouse as well, on a part of the upper colon. OXTR ligand binding was not present in OXTR KO mice (E, E’), followed by the post-processed cresyl violet tissue of the OXTR KO (F, F’).

Tables 1 and 2 summarize the statistical results. Due to the small sample sizes, males and females within each genotype were combined for our analyses. Multivariate analyses revealed a significant main effect of genotype [Pillai’s Trace = 0.875, *F*(8,16) = 13.96, *p* < 0.001], a significant main effect of competition dose [Pillai’s Trace = 0.983, *F*(16,34) = 2.052, *p* = 0.039], and a significant genotype × dose interaction [Pillai’s Trace = 1.005, *F*(16,34) = 2.146, *p* = 0.030] (Table 1). A significant genotype x dose interaction is consistent with specific OXTR ligand binding activity. All subsequently performed univariate tests assessed the effects of genotype and dose on individual regions of interest including all specimens, wild-type and knockout, in the final analysis (Table 2), with a specific interest in statistically significant genotype × dose interactions as an indication of true OXTR ligand binding. Bonferroni corrected p-values are reported. The primordial tooth measured at the incisors (Fig 1) showed a significant effect of genotype [*F*(1,23) = 33.303, *p* < 0.001], a significant effect of competition dose [*F*(2,23) = 14.100, *p* < 0.001], and a significant genotype × dose interaction[*F*(2,23) = 13.251, *p* = 0.001]. The ciliary body of the eye (Fig 2) showed a significant effect of genotype [*F*(1,23) = 39.823, *p* < 0.001], a significant effect of competition dose [*F*(2,23) = 8.563, *p* = 0.002], and a significant genotype × dose interaction [*F*(2,23) = 8.990, *p* = 0.001]. The whisker pads (Fig 3) showed a significant effect of genotype [*F*(1,23) = 4.659, *p* = 0.042], a trend toward an effect of competition dose [*F*(1,23) = 3.309, *p* = 0.055], and a significant genotype × dose interaction [*F*(2,23) = 8.277, *p* = 0.002]. The adrenal gland (Fig 4) showed a significant effect of genotype [*F*(1,23) = 16.612, *p* < 0.001], a significant effect of competition dose [*F*(2,23) = 4.6, *p* = 0.021], and a significant genotype × dose interaction [*F*(2,23) = 3.977, *p* = 0.033]. In mandibular and maxillary periodontium (Fig 5), a significant effect of genotype [*F*(1,23) = 6.043, *p* = 0.022], a significant effect of competition dose [*F*(2,23) = 4.934, *p* = 0.016], and a significant genotype × dose interaction was observed [F(2,23) = 4.771, *p* = 0.018]. In the anogenital region (Fig 6), a significant effect of genotype [*F*(1,23) = 5.805, *p* = 0.024], a significant effect of competition dose [*F*(2,23) = 4.042, *p* = 0.031], and a significant genotype × dose interaction was observed [F(2,23) = 3.485, *p* = 0.048].

**Fig 7:**
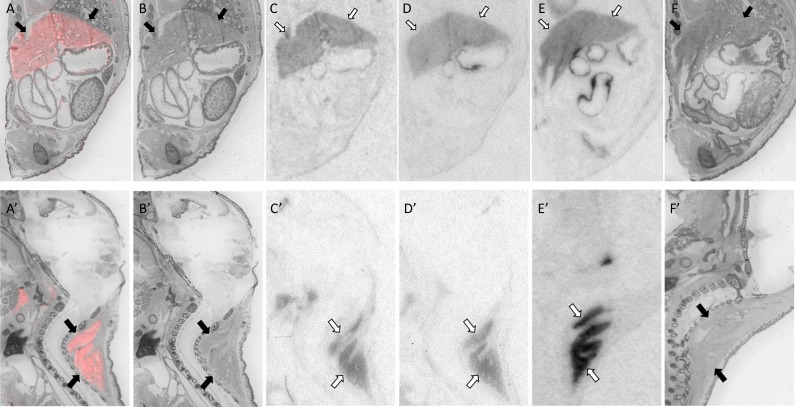
**Nonspecific binding in the liver (A-F) and brown adipose tissue (A’-F’) of a P0 male mouse.** Pseudo-color composite (OXTR in red, cresyl violet counterstain in gray) (A, A’), followed by cresyl violet counterstain (B, B’), with black arrows indicating region of interest. Nonspecific binding is indicated by white arrows in the 0 nM OXT competition (C, C’), followed by 1000 nM OXT competing with OVTA radioligand that did not reduce the signal (D, D’). Nonspecific binding was also present in OXTR KO mice (E, E’), followed by the post-processed cresyl violet tissue of the OXTR KO (F, F’).

The liver (Fig 7A–7F) and the brown adipose tissue (Fig 7A’–7F’) both exhibited non-specific binding. There were significant differences between genotype in both the liver [*F*(1,23) = 5.868, *p* = 0.024] and in the brown adipose tissue [*F*(1,23) = 12.416, *p* = 0.002], with OXTR KO mice showing *higher* density of radioactive ligand binding. Importantly, the dense film signal in either liver or brown adipose tissue was not competed off with excess unlabeled OXT in either WT or KO, as evidenced by the lack of a main effect of competition dose (liver [*F*(2,23) = 0.161, *p* = 0.852]; brown adipose [*F*(2,23) = 0.858, *p* = 0.437]) or a significant genotype × dose interaction term (liver [*F*(2,23) = 0.020, *p* = 0.98]; brown adipose [*F*(2,23) = 0.237, *p* = 0.791]).

**Table 1:** Multivariate test (MANOVA).

Variable	Levels	Pillai’s Trace	F-value (df)	p^a^
Genotype	2 (WT, OXTR KO)	0.875	13.96 (8,16)	<0.001
Dose	3 (0, 10, 1000 nM)	0.983	2.052 (16, 34)	0.039
Genotype × Dose	2 × 3	1.005	2.146 (16, 34)	0.030

^a^ Significance established at p<0.05.

**Table 2:** Univariate tests (ANOVA).

Region of Interest	Independent Variables	F-value (df)	p^b^
Tooth^a^	Genotype	*F*(1,23) = 33.303	< 0.001
	Dose	*F*(2,23) = 14.100	< 0.001
	Genotype × Dose	*F* (2,23) = 13.251	< 0.001
Eye^a^	Genotype	*F*(1,23) = 39.823	< 0.001
	Dose	*F*(2,23) = 8.563	0.002
	Genotype × Dose	*F* (2,23) = 8.990	0.001
Whisker pads^a^	Genotype	*F*(1,23) = 4.659	0.042
	Dose	*F*(2,23) = 3.309	0.055
	Genotype × Dose	*F* (2,23) = 8.277	0.002
Periodontium^a^	Genotype	*F*(1,23) = 6.043	0.022
	Dose	*F*(2,23) = 4.934	0.016
	Genotype × Dose	*F* (2,23) = 4.771	0.018
Adrenal gland^a^	Genotype	*F*(1,23) = 16.612	< 0.001
	Dose	*F*(2,23) = 4.600	0.021
	Genotype × Dose	*F* (2,23) = 3.977	0.033
Anogenital area^a^	Genotype	*F*(1,23) = 5.805	0.024
	Dose	*F*(2,23) = 4.042	0.031
	Genotype × Dose	*F* (2,23) = 3.485	0.048
Liver	Genotype	*F*(1,23) = 5.868	0.024
	Dose	*F*(2,23) = 0.161	0.852
	Genotype x Dose	*F*(2,23) = 0.020	0.980
Brown Adipose	Genotype	*F*(1,23) = 12.416	0.002
	Dose	*F*(2,23) = 0.858	0.437
	Genotype x Dose	*F*(2,23) = 0.237	0.791

^a^ Regions with a significant genotype x dose interaction term meet statistical criteria for specific OXTR ligand binding.

^b^ Bonferroni-corrected p-values. Significance established at p<0.05.

**Fig 8:**
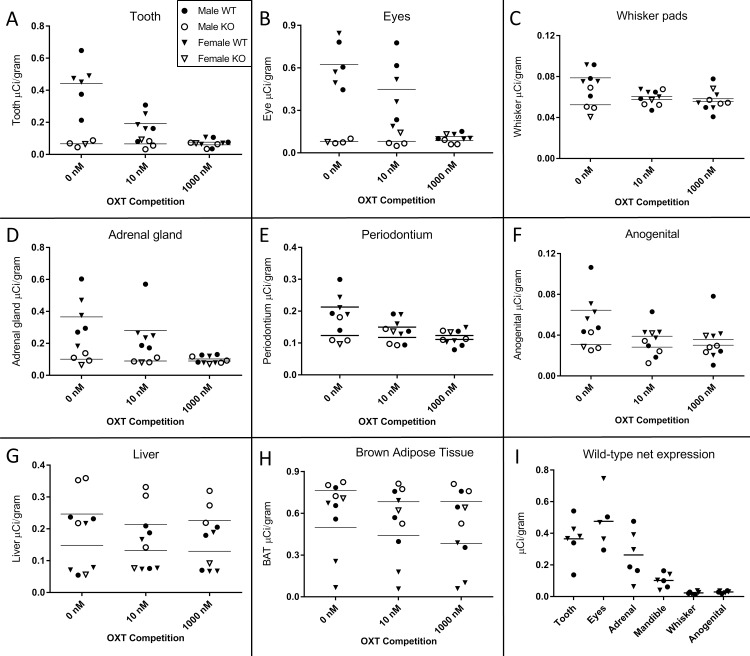
Quantification of receptor autoradiography for OXTR in peripheral tissue of wild-type and OXTR knockout mice. Differences between wild-type (WT) and knockout (KO) genotypes are evident in the 0 nM OXT competition condition, with displacement of binding in the WT as the competition increases across 10 nM and 1000 nM OXT. The y-axis of each graph has been fit to the scale of the data. (A) Primordial tooth; (B) Eyes; (C) Whisker pads; (D) Adrenal gland; (E) Periodontium; (F) Anogenital; (G) Liver; (H) Scapular brown adipose tissue. The net OXTR binding values (net = density of 0 nM- 1000 nM) for all regions in wild-type mice with specific OXTR ligand binding are compared against a common y-axis on the final panel (I). Group means and individual subject data are plotted (WT (filled symbols), OXTR KO (open symbols), males (circles), females (triangles)).

The results of the quantitative densitometry for each region of interest are presented in Fig 8. While males and females were analyzed together, they are graphed separately. Specificity in OXTR binding is detectable as a reduction in ligand binding with increasing doses of competition in the OXTR wild-type, with no change in densitometry across competition doses in the OXTR knockout.

## Discussion

Data within this paper provide evidence for the presence of OXTR in the periphery of the neonatal mouse, including tissues in the face, the oronasal cavity, the adrenal gland, and the anogenital area, with implications for development. The data in this report are consistent with prior evidence of the high specificity of this [^125^I]-OVTA ligand for OXTR in the brain and uterus across rodent species [19, 26–28]. Further, these data emphasize the value in competing off the ligand and comparing competitive ligand binding between WT and KO specimens in previously unconfirmed tissues. Using the OXTR KO as well as the competition with OXT, we were able to determine that some areas such as the liver and the brown adipose tissue, while they produce an intense ligand binding signal, do not appear to reflect robust OXTR levels. In addition to determining that the liver and brown adipose tissue do not contain robust levels of OXTR, despite the intense signal on the autoradiographic films, we were able to positively confirm specific OXTR signal in many areas (Fig 8I). Future studies are ongoing to investigate functionality and responsiveness to OXT in peripheral regions containing OXTR. Additionally, it is possible that genetic background, environmental factors, and sex differences may alter the levels of OXTR ligand binding observed here. This report is underpowered to detect modest sex differences and is not designed to test the potential for dynamic regulation of OXTR levels.

**Fig 9:**
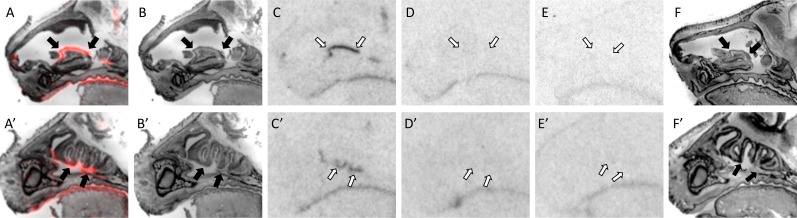
**OXTR ligand binding in the vomeronasal organ (A-F) and olfactory epithelium (A’-F’) of P0 mice.** Pseudo-color composite (OXTR in red, cresyl violet counterstain in gray) (A, A’), followed by cresyl violet counterstain (B, B’), with black arrows indicating region of interest. OXTR ligand binding is indicated by white arrows in the 0 nM OXT competition (C, C’), followed by 1000 nM OXT competing with OVTA radioligand to reduce the signal (D, D’). OXTR ligand binding was not present in OXTR KO mice (E, E’), followed by the post-processed cresyl violet tissue of the OXTR KO (F, F’).

While we obtained strong evidence for specific OXTR sites in the periphery, the areas we statistically quantified are not an exhaustive list of potential areas expressing OXTR. We selected areas with striking film density visible to the unaided eye after 72 hours of exposure to film and that were anatomically amenable to quantification. Small or anatomically ill-defined, faint, and frequently damaged areas were not included in our statistical analysis as presented in Figs 1–8 and Tables 1 and 2. For example, at 20 μm in 8 series, if a region is less than 480 μm in depth in the sagittal plane, then it may only be sampled 2 times and if a region is less than 320 μm, then it may only appear once in the series. Additionally, if a particular area is prone to tissue damage during sectioning, then this also reduces the sampling within an individual. We did not quantify the specific signal in the brain and spinal cord, since the focus of this research is on novel findings in the infant periphery. There are some areas that, while we did not quantify the film density, did appear to show specific OXTR ligand binding, with a signal in the WT that is absent in the KO and absent with 1000 nM OXT competition. Examples include the kidney (stippled ligand binding pattern below the adrenal gland in Fig 4C) and the nasal cavity (Fig 9), and in the brain and spinal cord. The ligand binding in the kidney and nasal cavity was similar to what we previously reported in E18.5 C57BL/6J embryos [19], and here we observed that the ligand binding was not apparent in the OXTR KO or with 1000 nM competition. Therefore, while not statistically analyzed, these results meet the same logic threshold used in our statistical analysis and often applied to tests of antibody specificity: there is a signal in the WT that is absent in the WT with competition and in the OXTR KO. There are other regions that we did not measure that would also likely contain OXTR. It is important to emphasize that we did not quantitatively measure or otherwise qualitatively assess other areas not listed. Therefore, unlisted regions have not been conclusively demonstrated to either contain or lack specific OXTR.

We measured two areas within the oral cavity which display prominent OXTR ligand binding: the primordial lower incisor region (tooth) and the oral periodontium. Both of these oral areas displayed significant differences in OXT receptor ligand binding between wild-type and knockout mice, with decreasing signal across competition doses in the wild-type mice, and a significant genotype x dose interaction. There is currently mixed evidence to support a hypothesis that OXTR in the neonatal oral cavity has developmental consequences for feeding related behavior. Prior characterization of the OXTR knockout mouse did not indicate any developmental differences in weight gain, and presumably feeding ability, of knockout pups [29]; however, *Magel2* deficient neonates are at risk for feeding dysfunction at birth, which is rescued by a single postnatal subcutaneous injection of OXT [30]. Intriguingly, the OXTR in the mouth of the neonatal mouse are conveniently positioned to potentially respond to OXT found in breast milk [31, 32]. The oral cavity and the nasal cavity are an interconnected space. In addition to the quantified binding in the oral cavity, we also obtained qualitative evidence of specific OXTR ligand binding in the nasal cavity (Fig 9). OXTR ligand binding in both the mouth and nose might be of particular interest given the rapid emergence of intranasal delivery of OXT in basic research and clinical trial settings. It has been argued that because intranasal delivery of OXT affects brain activity and behavior, that it must cross the olfactory epithelium and cribriform plate to reach the brain. However, the mechanisms of the brain and behavior effects of intranasal OXT are still unknown [33–36]. Perhaps there is some role for oronasal OXTR in the effectiveness of intranasal OXT delivery. Determining the expression of OXTR in the oronasal cavity of humans is warranted.

OXTR were observed in the eye, more specifically what appears to be in the region of the ciliary processes of the eye surrounding the iris. More precise techniques are needed to determine the structures exhibiting OXTR in detail, but candidate structures in this region are typically involved in muscle contractions regulating the lens and/or the pupil. Intranasal OXT treatment is associated with increased pupil dilation in adult human participants [37, 38]. Whether this effect is local action in the eye, centrally regulated, or both, remains to be determined.

The region around the whisker follicles of the whisker pads displayed significant differences between the wild-type and knockout genotypes. The significant genotype x dose interaction supports at least modest levels of OXTR in the whisker pads. A potential role for OXT at the OXTR in the whiskers remains unexplored. Developmental whisker deprivation significantly reduces production of OXT in the infant hypothalamus [39]. Whether OXTR in the whisker pads themselves provides feedforward or feedback regulation for this experience-dependent process is an intriguing possibility.

Outside of the facial region, OXTR expression was observed in the perineum of the anogenital area. The significant genotype by dose interaction supports specific OXTR in this area. This region is of particular interest due to prior research in rodents on the importance of anogenital licking and grooming in the infant from dams. Anogenital licking and grooming is necessary for the infant’s survival to aid in urination and bowel movements as their muscles develop [40, 41]. This maternal behavior appears to be elicited by chemosignals emitted from the preputial glands of the pup [42]. Rat pups that are more frequently licked and groomed presented attenuated stress responses persisting into adulthood [43, 44]. “Licking-like tactile stimulation” of the anogenital region of neonatal rats activates oxytocinergic neurons in the hypothalamus and produces an increase in spinal OXT [45]. It would be of interest to determine if salivary transmission of maternal OXT to infant OXTR in the anogenital area alters the response to licking-like tactile stimulation.

We confirmed selective OXTR ligand binding in the neonatal adrenal gland. OXT peptide was first identified in the adrenal gland in comparison to relative levels of arginine vasopressin [46]. OXT may act in the adrenal gland as an attenuating signal to stress, inhibiting cortisol release [47] and modulating several other indirect pathways [48]. OXTR knockout mice display significantly lower levels of adrenaline in comparison to wild-type mice, suggesting OXT plays an important role in regulating sympathetic tone via the adrenal gland [49].

Genotype differences were observed in the liver and brown adipose tissue; however, because the binding was denser in the OXTR KO neonate, and binding was not competed off in either the WT or KO with excess unlabeled OXT, these data are consistent with non-specific binding and are unlikely to represent a robust OXTR signal in these tissues. It is possible that some small portion of the binding was truly ligand binding to OXTR, but if present, it was not detectable with this sample size. It is the authors’ belief that the intense non-specific signal in liver and brown adipose tissue may be an artifact of nonspecific binding to lipid-dense tissues. It is intriguing that the KO seems to show more of this non-specific binding. If this is due to more lipid content in the KO, it will be important to test in the future if this is a precursor to the established metabolic disorder that emerges later in life for OXTR KO mice [29]. As previously mentioned, developing juvenile OXTR knockout mice do not differ in weight from wild-type mice, however adult OXTR knockout mice are significantly heavier in overall body weight than their wild-type littermates after 12 weeks of age [29]. These differences have been observed in OXT peptide knockout mice as well, except females also presented the obesogenic phenotype, which was not present in OXTR knockout females [49]. The overall differences in weight and adiposity could not be attributed to chow intake nor basal levels of activity, suggesting a disruption in cellular metabolism. Histology of brown adipose tissue from OXTR knockout mice suggested hypoactivity, as the brown adipocytes were composed of a single large lipid droplet, a structure more similar to white adipocytes, than the multilocular structure of normal brown adipocytes [29]. The data presented within this paper suggest a new hypothesis that lipidogenic differences are apparent in the brown adipose tissue and liver adipocytes between knockout and wild-type OXTR mice at birth.

Infant OXT can be modulated during very early development by sensory experience [39], and social experience, primarily parental care [50–54]. The mouse brain undergoes robust changes in OXTR expression during early prenatal [55] and postnatal development [19], and the presence of a dynamic process appears to be conserved across species, although the precise details are species-specific [56]. In mice, the postnatal data show significant differences across ages in regions of the brain pertaining to social and sensory development such as the hippocampus, lateral septum, and neocortex. Integration of sensory and social experience in the postnatal environment begins very early, with infants learning associations with stimuli within hours of birth, in spite of immature sensory systems [57–61]. OXT is emerging as a probable candidate in the coordination of sensory and social learning in the developing brain [12], and data within this paper supports further research into the role of OXT via peripheral OXTR as a modulator of social contact-dependent development in the socially naïve neonate.

## Supporting information

S1 Table Densitometry data for all quantified areas. (XLSX)
